# Political Myopia

**Published:** 1882-04

**Authors:** F. P. Porcher


### POLITICAL MYOPIA.

Legislators (we speak impersonally) will vote supplies for a good many different and indifferent objects; but, for others with apparently remote advantages—such, for example, as the preservation of health—before deciding, the sanitary fat (as Surg. Billings observes) will keep up for a time a mighty sizzling! The short-sighted selfishness of men—what may be termed a political *myopia*—the conflicts of narrow, local interests, the injury to the trade and gain of the few who are interfered with for the benefit of the many, the proneness to procrastinate—specially where money is to be paid out, and because it is notorious that a man will often devote for the security and advantage of his property, his dependents, and even for his animals, what he would hesitate to sacrifice for the preservation of the life of his family; a natural, and sometimes a prudent spirit of economy—all these tend to obstruct and delay the adoption of measures of the very highest utility. Men are unwilling to diminish their comfort, their business, or their earnings; so it is even possible that through the self-satisfied ignorance, the obstinacy, and the penny-wise and pound-foolish fatuity of some, in a single locality 10,000 days in a year are lost by sickness, and 1000 preventable deaths occur! Such is the strange, mixed web of human motives and actions. —*Dr. F. P. Porcher, in Address to Legislature of South Carolina, on "The Sanitary Needs of the State."*

